# A digital mRNA expression signature to classify challenging Spitzoid melanocytic neoplasms

**DOI:** 10.1002/2211-5463.12897

**Published:** 2020-06-05

**Authors:** Lisa M. Hillen, Milan S. Geybels, Ivelina Spassova, Jürgen C. Becker, Thilo Gambichler, Marjan Garmyn, Axel zur Hausen, Joost van den Oord, Véronique Winnepenninckx

**Affiliations:** ^1^ Department of Pathology GROW‐School for Oncology and Developmental Biology Maastricht University Medical Center (MUMC+) Maastricht the Netherlands; ^2^ Department of Epidemiology GROW School for Oncology and Developmental Biology Maastricht University Maastricht the Netherlands; ^3^ Department for Translational Skin Cancer Research (TSCR) German Cancer Consortium (DKTK) University Hospital Essen Essen Germany; ^4^ Deutsches Krebsforschungsinstitut (DKFZ) Heidelberg Germany; ^5^ Department of Dermatology Ruhr‐University Bochum Bochum Germany; ^6^ Laboratory of Dermatology Department of Oncology and Department of Dermatology University Hospitals Leuven University of Leuven KUL Leuven Belgium; ^7^ Department of Pathology University Hospitals of Leuven University of Leuven KUL Leuven Belgium; ^8^ Laboratory Translational Cell and Tissue Research University of Leuven, KU Leuven Belgium

**Keywords:** atypical Spitz nevus, gene expression profiling, malignant Spitz tumor, molecular signature, mRNA, Spitz nevus

## Abstract

Spitzoid neoplasms are a challenging group of cutaneous melanocytic proliferations. They are characterized by epithelioid and/or spindle‐shaped melanocytes and classified as benign Spitz nevi (SN), atypical Spitz tumors (AST), or malignant Spitz tumors (MST). The intermediate AST category represents a diagnostically challenging group since on purely histopathological grounds, their benign or malignant character remains unpredictable. This results in uncertainties in patient treatment and prognosis. The molecular properties of Spitzoid lesions, especially their transcriptomic landscape, remain poorly understood, and genomic alterations in melanoma‐associated oncogenes are typically absent. The aim of this study was to characterize their transcriptome with digital mRNA expression profiling. Formalin‐fixed paraffin‐embedded samples (including 27 SN, 10 AST, and 14 MST) were analyzed using the NanoString nCounter PanCancer Pathways Panel. The number of significantly differentially expressed genes in SN vs. MST, SN vs. AST, and AST vs. MST was 68, 167, and 18, respectively. Gene set enrichment analysis revealed upregulation of pathways related to epithelial–mesenchymal transition and immunomodulatory‐, angiogenesis‐, hormonal‐, and myogenesis‐associated processes in AST and MST. A molecular signature of SN vs. MST was discovered based on the top‐ranked most informative genes: *NRAS*,* NF1*,* BMP2*,* EIF2B4*,* IFNA17*, and *FZD9*. The AST samples showed intermediate levels of the identified signature. This implies that the gene signature can potentially be used to distinguish high‐grade from low‐grade AST with a larger study cohort in the future. This combined histopathological and transcriptomic methodology is promising for prospective diagnostics of Spitzoid neoplasms and patient management in dermatological oncology.

## Graphical abstract

Spitzoid neoplasms are cutaneous melanocytic proliferations and classified as benign Spitz nevi (SN), atypical Spitz tumors (AST), or malignant Spitz tumors (MST). In particular, AST are challenging since on histopathological grounds, their benign or malignant potential is unpredictable. We characterized their transcriptome with digital mRNA expression profiling. A molecular signature was discovered, which might be promising for diagnostics in dermatological oncology.

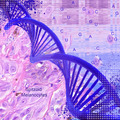

**Abbreviations**
AST: atypical Spitz tumor
BMP: bone morphogenetic protein
CV: cross‐validation
EIF2: eukaryotic initiation factor 2
FDR: false discovery rate
FFPE: formalin‐fixed paraffin‐embedded
FZD: frizzled class receptor
GSEA: gene set enrichment analysis
H.E.: hematoxylin and eosin
IFN: interferon
LASSO: least absolute shrinkage and selection operator
MAPK: mitogen‐activated protein kinase
MSigDB: Molecular Signatures Database
MST: malignant Spitz tumor
MUMC+: Maastricht University Medical Center Plus
NCN: nevocellular nevi
NF1: neurofibromin 1
NRAS: neuroblastoma RAS viral (v‐ras) oncogene homolog
PI3K: phosphatidylinositol 3'‐kinase
SKCM: skin cutaneous melanoma
SMAD: small mothers against decapentaplegic
SN: Spitz nevi
STUMP: Spitzoid tumor of uncertain malignant potential
T: tumor
TCGA: The Cancer Genome Atlas
UCSC: University of Santa Cruz
WIF1: Wnt inhibitory factor 1

Spitzoid melanocytic neoplasms comprise uncommon melanocytic skin lesions usually arising in children and adolescents, but may also occur in older individuals [1]. Key challenges at the time of primary histopathological evaluation of Spitzoid melanocytic neoplasms are the distinction between benign or malignant processes and the choice of optimal therapy in case of melanocytic atypia or malignancy. Spitzoid neoplasms were first described by the American pathologist Sophie Spitz in 1948 as ‘juvenile melanomas’ or as ‘melanomas of childhood’ [2] and are composed of large epithelioid and/ or spindle‐shaped melanocytes with large nuclei that contain vesicular chromatin and often prominent nucleoli [3]. They may be purely junctional, compound, or dermal, and have a background with variable amounts of lymphocytes, blood vessels, and sclerosis.

According to the recently launched fourth edition of the World Health Organization classification of skin tumors, these lesions range from benign Spitz nevi (SN) to highly proliferative and pleomorphic malignant Spitz tumor (MST) or Spitzoid melanoma [4]. Complicating the diagnostic issue, the major challenge lies in a subset of lesions that fall in between the two extreme types, that is, the atypical Spitz tumor (AST) or Spitzoid tumor of uncertain malignant potential (STUMP, [4, 5). The biological potential of AST/STUMP is currently impossible to predict on histopathological grounds alone [6, 7, 8.

The to‐date identified molecular alterations in these lesions have not yet resulted in one stand‐alone or a clearly defined distinct set of ancillary biomarkers that can reliably predict whether an ambiguous Spitzoid neoplasm has benign or malignant potential (for a review, see Ref. [9, 10, 11). Consequently, potentially benign AST may be misdiagnosed as MST, thereby exposing the patient to aggressive surgical and oncological treatment. Until now, only few transcriptomic studies on Spitzoid melanocytic lesions have been performed [12, 13, 14. Advancing in that direction, we recently identified a distinct molecular expression profile differentiating SN from common nevocellular nevi (NCN), thereby suggesting a differential pathogenesis of the two groups of melanocytic neoplasms [15].

Based on these findings, we wanted to know whether such a differential gene expression profile can be found along the whole spectrum of Spitzoid melanocytic neoplasms (SN vs. AST vs. MST), and whether such a profile might be of help in the differential diagnosis in daily histopathology practice, clinical treatment decisions, and patient management. The aim of this study was thus to characterize the transcriptomic landscape of SN, AST, and MST with digital mRNA expression profiling. Our research question was threefold: (a) Are there differentially expressed genes in the three categories of Spitzoid melanocytic neoplasms (AST vs. MST, SN vs. AST, and SN vs. MST)? (b) Do possibly identified mRNA transcripts belong to distinct gene pathways?; And (c) can a distinct gene expression signature of top‐ranked genes be identified to discriminate benign from malignant lesions?

## Materials and methods

### Patients and tissues

Formalin‐fixed paraffin‐embedded (FFPE) excision specimens of patients harboring a SN, an AST, or a MST were retrieved from the archives of the Department of Pathology at the University of Leuven, KUL Belgium, the Maastricht Pathology Tissue Collection from the Department of Pathology, Maastricht University Medical Center, (MUMC+), the Netherlands, and the Department of Dermatology, Ruhr‐University Bochum, Bochum, Germany. The patients kindly provided their written informed consent at hospital admission to the processing of tissue and personal data. All samples had been excised for diagnostic and therapeutic reasons. All use of tissue and patient data was in agreement with the Dutch Code of Conduct for Observational Research with Personal Data (2004) and Tissue (2001) and in accordance with Ethical Principles for Medical Research Involving Human Subjects (World Medical Association Declaration of Helsinki). Histopathological diagnoses were previously defined in routine diagnostics and were confirmed by three experienced dermatopathologists (VW, JVO, and LMH).

**Fig. 1:**
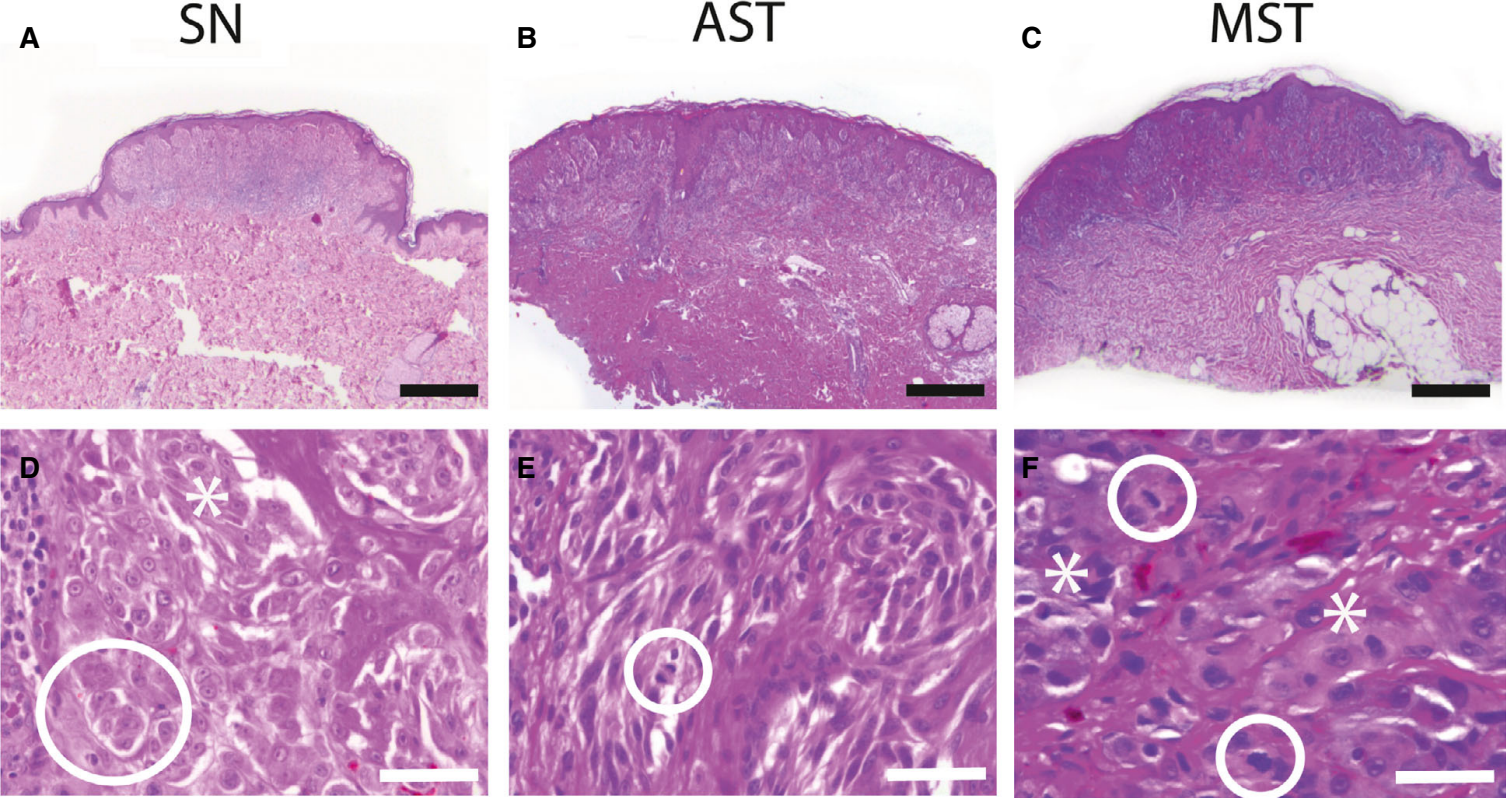
Histomorphology of a prototypic SN, AST, and a MST. (A) Histology of a compound SN used in this study with a dome‐shaped configuration. H.E. staining, length of scale bar: 500 µm. (B) Illustration of an AST investigated in this study with a compound architecture. The lesion is slightly asymmetrical, and there is epidermal hyperplasia. H.E. staining, length of scale bar: 500 µm. (C) Morphology of a MST from the MST cohort. H.E. staining, length of scale bar: 500 µm. (D) Higher magnification of a SN depicted from (A) showing the two cell types of Spitzoid melanocytes with epithelioid Spitzoid morphology (encircled) with abundant eosinophilic ground‐glass cytoplasm containing vesicular nuclei with prominent nucleoli and spindle‐shaped Spitzoid melanocytes (asterisk). There are retraction clefts around the melanocytic cell nests. H.E. staining, length of scale bar: 100 µm. (E) Magnification of an AST from (B) showing nests of Spitzoid melanocytes with hyperchromatic nuclei and increase in N/C ratio. Spindle‐shaped melanocytes dominate in relation to epithelioid cells. A mitotic figure (encircled) is localized in the superficial area of the lesions at the epidermal junction, length of scale bar: 100 µm. (F) Higher magnification from of a MST depicted from (C) with frequent irregular mitotic figures (encircled), which are localized in the dermal depth of the lesion. There is strong cytonuclear atypia (asterisks) with irregular nuclear configuration, aberration of the nucleus‐to‐cytoplasm ratio, and hyperchromasia, length of scale bar: 100 µm.

A total of 51 cases were included in the analysis. Of these, 27 were histologically diagnosed as banal SN, 10 as AST, and 14 as MST. SN were defined as benign melanocytic nevi composed of large epithelioid, oval, or spindled melanocytes arranged in nests and/ or fascicles without significant cytonuclear atypia. The diagnosis of an AST was made if there was an increase in at least one worrisome histological feature, that is, ulceration, size > 5 mm, increase in cell density with confluent growth, infiltrative growth into subcutaneous tissue with ‘pushing’ margins, increased cytonuclear atypia, > 2 dermal mitoses, absence of junctional clefts, few or no Kamino bodies, and more extensive pagetoid extension. The diagnosis of MST was based on the following histopathological criteria: ulceration, asymmetrical architecture, infiltrative growth, severe and/or confluent cytonuclear atypia, frequent dermal mitoses, pushing borders, epidermal effacement, and pagetoid extension [4]. Figure 1 illustrates the morphology of a prototypic SN (Fig. 1A,D), AST (Fig. 1B,E), and MST (Fig. 1C,F). The study was approved by the Maastricht Ethics Committee of the University of Maastricht, the Netherlands, and by the Institutional Review Board of the University Hospitals of Leuven, Belgium (project number S 59659).

### Patient cohort

The SN group consisted of 27 patients with 15 females and 11 males ranging in age between 2 and 62 years (mean 31.3 years, median 31.5 years; Table S1); from one case (SN23), clinical data were not available. Histologically, nine SN were diagnosed as dermal, five SN as junctional, and 13 as compound lesions. SN4 showed dysplastic features with mild cytonuclear atypia and variation in cellular morphology but did not fulfill criteria for the diagnosis of an AST. The AST group consisted of 10 patients, with seven female and three male patients showing a similar age distribution ranging from 9 to 55 years (mean 31.3 years, median 29 years; Table S1). The AST group consisted of two dermal and eight compound lesions. Like the SN group, the AST group showed localization on the extremities, trunk, and head and neck area. A sentinel node procedure was not performed in the AST cohort in accordance with the wishes of the patients.

The MST group consisted of 14 cases with nine females and five males ranging in age from 20 to 66 years (mean 53.1 years, median 26.5 years; Table S1). The MST group showed a mean Breslow thickness of 1.39 mm (range from 0.3 to 2.98 mm, median 1.27 mm). Three patients showed regional lymph node metastasis (MST2, MST4, and MST9) with in two of them development of distant metastasis during follow‐up (MST2 and MST4).

Follow‐up was available for all patients except for cases SN23, AST1, AST2, and AST10 (Table S1).

### Tissue preparation and mRNA expression analysis

Serial sections (5 µm) were cut from FFPE tissue blocks. One section per sample was mounted onto a glass slide and stained with hematoxylin and eosin (H.E.) to reassure that at least 70% of the section consisted of melanocytic cells. For RNA isolation, FFPE sections were collected in Eppendorf tubes, deparaffinized with xylol washing steps (1–4 times), and rehydrated in a graded alcohol series, starting at 100%, proceeding with 96%, and completing with 70% EtOH. In between each step, tubes were washed with xylol/EtOH in a centrifugation step for 5 min at 13 000 ***g***. Subsequently, the pellets were dried for 1 h at 56 °C. RNA was isolated by AllPrep DNA/RNA FFPE Kit (Qiagen, Hildesheim, Germany) according to the manufacturer's instructions. Purified RNA was measured in a spectrophotometer (NanoDrop 2000; Thermo Scientific, Breda, the Netherlands). Finally, another section was cut from the rest of the FFPE block and stained with H.E., and the inclusion criteria were evaluated again on this section. Gene expression profiling was performed with the NanoString nCounter Gene Expression Platform (NanoString Technologies, Center of Medical Biotechnology, University Duisburg/ Essen, Germany). The methodology detects the expression of up to 770 genes (including 40 housekeeping genes) from 13 canonical pathways including Hallmark cancer genes (see PanCancer Pathways Panel: https://www.nanostring.com/products/gene‐expression‐panels/hallmarks‐cancer‐gene‐expression‐panel‐collection/pancancer‐pathways‐panel). The mRNA array data are deposited in the NCBI’s Gene Expression Omnibus database (accession number: GSE139314).

### Bioinformatic and statistical analysis

#### Data normalization

Gene expression counts were normalized based on total library size (total number of counts per sample; Fig. S1 and Table S3). As such, both the housekeeping and endogenous genes were used for normalization. This method is commonly used when many genes are analyzed simultaneously (NanoString recommends > 300). The studied samples showed a mean library size of 307 914 counts [median 292 412; range 789 054 (sample AST4) to 19 042 (sample MST4)]. All analyses were performed using R and Bioconductor packages.

#### Identification of differentially expressed genes

Marker discovery and validation of differentially expressed genes from the nCounter data was done using the *limma* and *voom* method in R [16]. Three conditions were studied: (a) SN vs. MST, (bb) SN vs. AST, and (c) AST vs. MST. The *limma* procedure borrows information across all genes, which makes the analysis robust even when the sample size is small [17]. The *voom* procedure was applied to nonparametrically estimate the mean–variance relationship of the log‐transformed gene counts, which is important when dealing with expression count data [18]. This procedure takes as input the raw counts, and total library size (total number of counts) per sample is used as a scaling factor to normalize the counts (Table S3). The *voom* procedure generates precision weights, which are incorporated into the *limma* linear modeling procedure, and this removes the mean–variance relationship in the log‐counts. The normalized log‐counts were used in subsequent analyses (Fig. S2). To control for multiple testing, an adjusted *P*‐value [i.e., false discovery rate (FDR) *q*‐values] threshold of 25% was used for statistical significance. A heatmap of the expression data was generated using *pheatmap* in R. The data were scaled and centered before plotting using *scale* in R.

#### Identification of a gene signature

The Least Absolute Shrinkage and Selection Operator (LASSO) procedure was applied in R to create a gene expression signature for classifying SN in contrast to MST that was based on a small subset of the most informative gene expression markers. To prevent overfitting, all the genes were used as input for the analyses. A 10‐fold cross‐validation (CV) and the binomial deviance criterion were used to identify the optimal value for the tuning parameter that resulted in the smallest binomial deviance for classifying. In order to generate a robust gene expression signature, the LASSO procedure was run 500 times with each time using a randomly selected CV split of the data. Genes that were selected in at least 250 of the 500 LASSO individual models were included in the final signature. Each gene’s average model coefficient (or relative weight in the signature) across all repetitions was calculated. Using this method, a model of six top‐ranked gene expression markers was identified where every marker had a corresponding LASSO coefficient to distinguish SN from MST. A sum of the expression weights of the six top‐ranked gene expression markers was calculated per sample and evaluated in relation to clinical and histological parameters.

A genetic dataset for skin cutaneous melanoma (SKCM) provided by The Cancer Genome Atlas (TCGA, [19]) was downloaded from University of Santa Cruz (UCSC) Xena genome browser (https://xenabrowser.net/datapages/?cohort=TCGA%20Melanoma%20(SKCM)&removeHub=https%3A%2F%2Fxena.tre), providing mutational data for 473 melanoma samples. Mean expression values and *P*‐values (> 0.05) were calculated using a *t*‐test for each of the top‐ranked signature genes in primary SKCM cases and metastatic samples.

### Pathway analysis

A gene set enrichment analysis (GSEA) was done using the camera method (*limma* in R) to identify pathways that are significantly up‐ or downregulated in the three studied contrasts [20]. A matrix of gene expression levels (No. genes = 770) in the different samples was used as input for the analysis. An advantage of this method is that it accounts for intergenomic correlation. The gene expression data were compared to the HALLMARK Molecular Signatures Database (MSigDB, Broad Institute; No. = 50). The gene set was downloaded from: http://bioinf.wehi.edu.au/software/MSigDB/.

## Results

### Differential gene expression in the three categories of Spitzoid melanocytic neoplasms

**Fig. 2:**
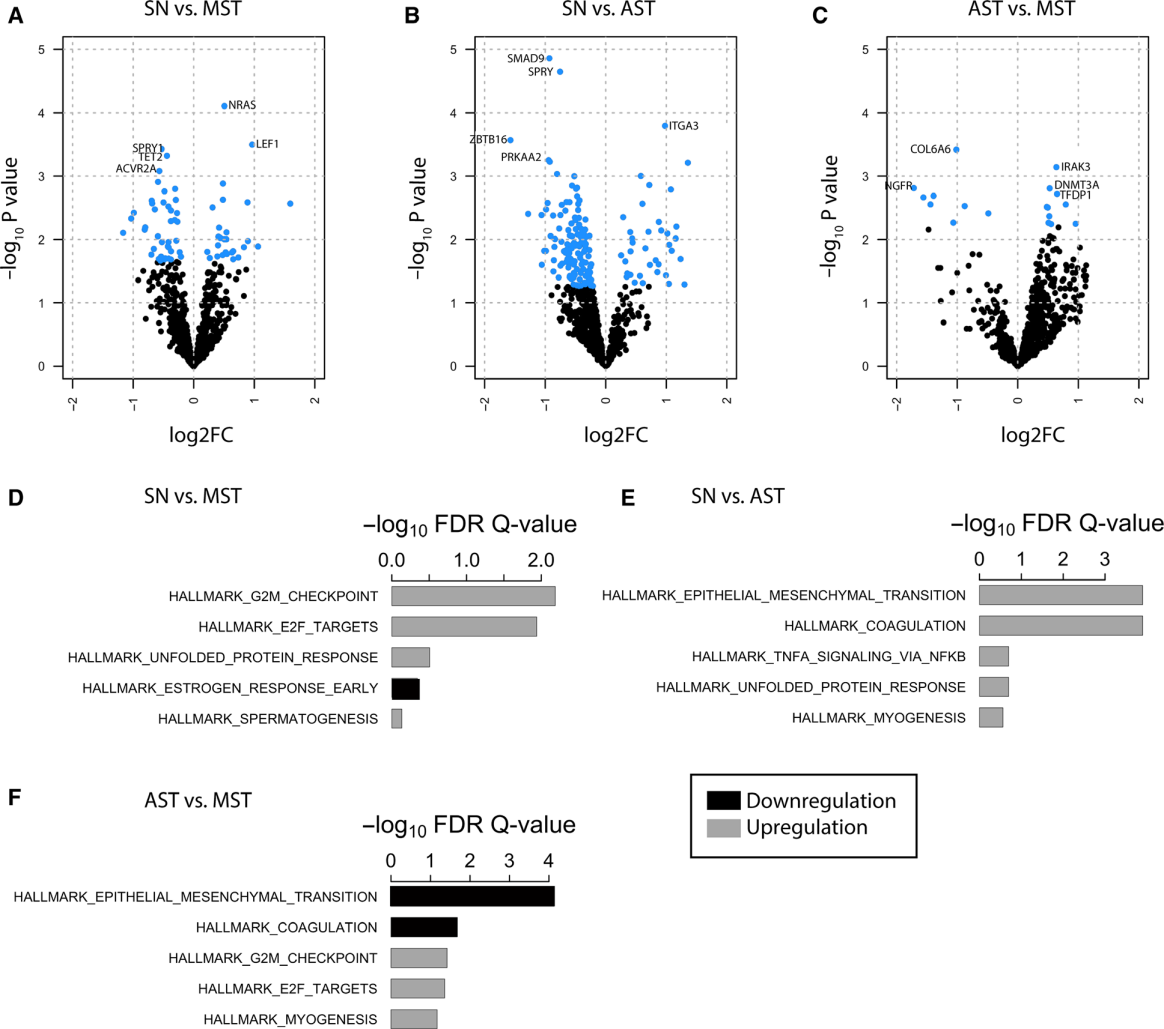
(A–C) Volcano plots identifying the differentially expressed genes in the three studied conditions: SN vs. MST (A), SN vs. AST (B), and AST vs. MST (C). The *x*‐axis shows the absolute log fold change (log 2FC), and the *y*‐axis represents the ‐log_10_ of the *P*‐value, thereby indicating statistically significantly differentially expressed genes. Each dot in the scatter represents a gene, with significant genes colored in blue. There were 68 genes differentially expressed in SN in relation to MST (*P*‐value < 0.05), of which 27 had a higher mean expression level (positive log FC), and 41 genes had a lower mean expression level [negative log FC, (A)]. In the SN vs. AST group, there were 167 differentially expressed genes (B). Out of these, 36 genes showed a positive log FC and 131 had a negative log FC. In the AST vs. MST group, there were 18 differentially expressed genes with 10 transcripts showing a positive log FC and eight transcripts showing a negative log FC (C). The top five differentially expressed genes are labeled with gene names. The other differentially expressed gene names are provided in Table S2 in the supplemental material. (D–F) The identified top‐five‐ranked gene pathways are listed with corresponding –log_10_ of the FDR *Q*‐value. Results of the Hallmark gene sets (including 50 gene sets), which were extracted from the MSigDB are shown for the three studied groups, that is, SN vs. MST (D), SN vs. AST (E), and AST vs. MST (F). Gray bars indicate upregulation, and black bars show downregulation of the gene pathways; for detailed characteristics of identified gene pathways, see also supplemental data, Table S4.

Initially, we investigated whether there are differentially expressed genes in the three groups of Spitzoid melanocytic neoplasms. The number of significantly differentially expressed genes (a) SN vs. MST, (b) SN vs. AST, and (c) AST vs. MST was 68, 167, and 18, respectively. The gene transcripts are detailed in Table S2 for the three studied conditions. For each identified gene, the log FC value, the average expression of the normalized log_2_ intensity value from *limma*, the *P*‐value, the t‐value, and the adjusted *P*‐value (or FDR *q*‐value) used to correct for multiple testing are given. Volcano plots of the results from the three studied contrasts are shown in Fig. 2A‐C. The blue dots indicate the significantly differentially expressed gene transcripts with the top five genes labeled with their gene names.

#### Differential gene expression in SN vs. MST

Sixty‐eight out of the 770 genes showed significantly differential expression in the SN vs. the MST group (*P*‐value < 0.05; Table S2.1). In the SN group, 27 transcripts had a higher mean expression level with a positive log FC and 41 transcripts showed a lower mean expression level with a negative log FC when compared to the MST group (Fig. 2A). The gene transcript with the lowest log FC (log FC = −1.17) was *WIF1*. The transcript *SPP1* showed the highest log FC (log FC = 1.59). An absolute log FC of at least 1 was identified in the four gene transcripts *LAMB4*,* SPP1*,* PLA1A*, and *WIF1* (Table S2.1).

#### Differential gene expression in SN vs. AST

In the group of SN vs. AST, 167 genes out of the 770 genes showed differential expression. From these, 36 genes showed a positive log FC and 131 had a negative log FC. Figure 2B shows the volcano plots with the identified differentially expressed genes in SN vs. AST. The lowest log FC (log FC = −1.57) was seen for the transcript *ZBTB16*, whereas the highest log FC (log FC = 1.36) was identified for *RXRG* (Table S2.2). Out of the 167 genes, 15 gene transcripts showed an absolute log FC of 1. The gene transcripts *ZBTB16*,* FGF14*,* SGK2*,* WT1*,* and CNTFR* had a log FC < −1, and the gene transcripts *ITGB3*,* PLA2G2A*,* SHC4*,* FOSL1*,* NGFR*,* SOCS3*,* IL13RA2*,* COMP*,* NTRK1*, and *RXRG* showed a log FC > 1 (Table S2.2).

#### Differential gene expression in AST vs. MST

In the AST vs. MST cohort, there were 18 differentially expressed genes with 10 transcripts showing a positive log FC and 8 transcripts with a negative log FC (range −1.72 to 0.96; Fig. 2C, Table S2.3). *NGFR*,* PLA2G2A*,* COL11A1*,* ITGA3*,* ITGB8*, and *COL6A6* were downregulated with a log FC < −1. The *FGF19* transcript had the highest log FC (log FC = 0.96), and *NGFR* showed the lowest log FC (log FC = −1.72). None of the 18 identified transcripts showed a log FC> 1.

### Pathway analysis

Gene set enrichment analysis was done to identify up‐ and downregulated pathways in the three studied groups of Spitzoid melanocytic lesions using the camera method. Out of the 50 investigated MSigDB gene sets from Hallmark, a top of ten gene pathways was identified. The MSigDB gene sets are included in the following broad categories: (a) cell cycle/ proliferation; (b) cytokine/ immune/ inflammatory; (c) matrix/ adhesion; (d) hormone/ receptor/ signal transduction; and (e) transport and other. Detailed characteristics of the identified gene pathways including number of corresponding genes, direction of regulation, *P*‐values, and FDR *q*‐values are given in the supplemental data (Table S4) and Fig. 2D‐F. A stringent FDR *q*‐value threshold of 0.05 was used for statistical significance. As such, the number of significant pathways for SN vs. MST was two with a FDR *q*‐value of 0.0071 for the Hallmark G2M checkpoint pathway and a FDR *q*‐value of 0.0124 for the E2F targets pathway. There was downregulation of the Hallmark estrogen response early pathway and upregulation of the Hallmark spermatogenesis pathway in SN vs. MST (Fig. 2D). In the group of SN vs. AST, there was significant upregulation of the Hallmark epithelial–mesenchymal transition pathway (FDR *q*‐value = 0.0001) and the Hallmark coagulation pathway (*P* = 0.0001; Fig. 2E). For the AST vs. MST condition, there were four significant pathways including the Hallmark epithelial–mesenchymal transition pathway (FDR *q*‐value = 0.0001), the Hallmark coagulation pathway (FDR *q*‐value = 0.0209), the Hallmark G2M checkpoint pathway (FDR *q*‐value = 0.0406), and the Hallmark E2F targets pathway (FDR *q*‐value = 0.0468; Fig. 2F).

Interestingly, in SN vs. AST there was significant upregulation of the Hallmark epithelial–mesenchymal transition pathway and the Hallmark coagulation pathway, whereas in AST vs. MST, these pathways were significantly downregulated. The same trend was seen for the Hallmark myogenesis pathway, the Hallmark angiogenesis pathway, and the Hallmark complement pathway with upregulation in the group of SN vs. AST and downregulation in the group of AST vs. MST. However, the different regulation of these pathways failed to be statistically significant.

### Identification of a molecular gene expression signature

**Fig. 3:**
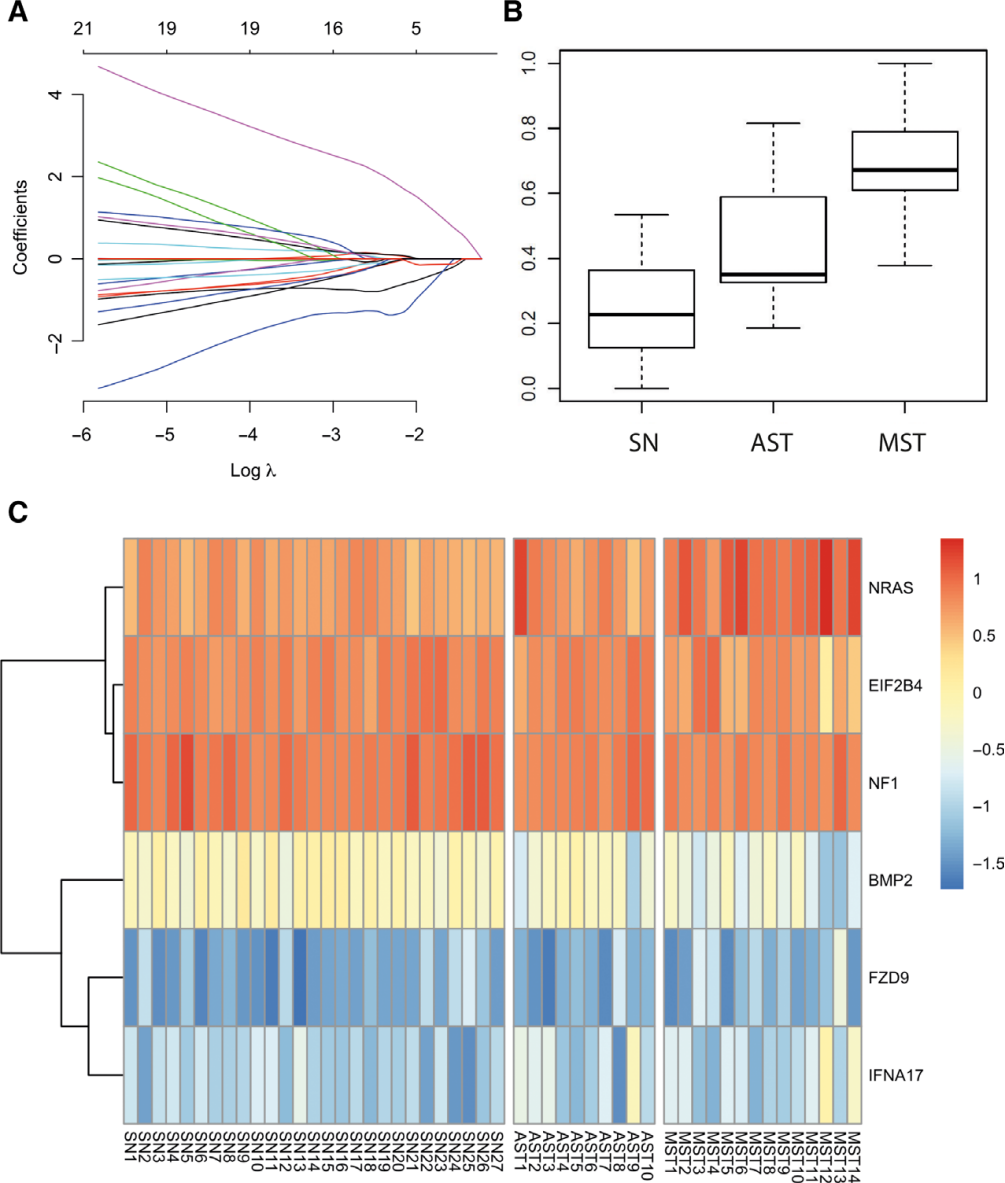
(A) Coefficient path from LASSO regularization for classifying. The colored lines represent individual genes. The ordinate shows the value of the coefficients associated with each gene as a function of log λ. A 10‐fold CV identified the value for the tuning parameter that resulted in the lowest binomial deviance for classifying with a model of six top‐ranked gene expression markers. (B) Boxplots of signature levels (range 0–1) in the group of SN (left), AST (center), and MST (right). The AST group shows intermediate levels of the signature in between SN and MST. (C) Supervised heatmap of the top six validated most informative gene transcripts which were included in the signature. The samples are grouped by patients harboring a SN, an AST, or a MST; that is, Spitzoid melanoma. Each column represents a patient sample from the three groups of Spitzoid melanocytic neoplasms. The rows of the heatmap are the identified signature genes *NRAS*,* EIF2B4*,* NF1*,* BMP2*,* FZD9*, and *IFNA17*. The highest expression levels are shown in red staining, and lowest expression levels are shown in blue staining, yellow color represents intermediate expression (figure legend). The rows were clustered based on Euclidean distance and the complete linkage method.

The statistical learning method LASSO was used to create a molecular profile of the SN vs. MST group. From the analysis, a final gene expression signature was derived. A 10‐fold CV identified the value for the tuning parameter that resulted in the lowest binomial deviance for classifying (Fig. 3A). The gene signature included six top‐ranked gene transcripts: *NRAS*,* EIF2B4*,* NF1*,* BMP2*,* FZD9*, and *IFNA17* (Fig. 3C). Detailed characteristics of the identified signature genes are summarized in Table 1 with chromosomal location, target sequence, subcellular localization, function of the target protein, and cellular pathway mapping. The signature levels of each sample are given as a multiplication from the gene expression level of the six genes with the associated LASSO coefficients (Table 1, last column). The gene transcripts *NRAS*,* IFNA17*, and *FZD9* were upregulated, and *EIF2B4*,* BMP2*, and *NF1* were downregulated in the SN group compared to the MST group.

**Table 1:** Characteristics of the 6 top‐ranked gene transcripts in SN vs. MST. HH, hedgehog; JAK‐STAT, Janus kinase/ signal transducers and activators of transcription (JAK/STAT) pathway; LASSO, coefficients used to calculate the signature; PI3K, PI3K‐Akt signaling pathway; RAS, Ras proteins are GTPases that function as molecular switches for signaling pathways; TGF‐B, transforming growth factor‐beta (TGF‐beta).

Gene and chromosomal location	Reference sequence ID and target sequence	Protein descript‐ion	Subcellular locus of protein	Function	Gene to cellular pathway mapping	LASSO coefficient (weight)
*NRAS*, neuroblastoma RAS viral (v‐ras) oncogene homolog, Chr. 1	NM_002524.3 ACCCTGGTCCTGACTTCCCTGGAGGAGAAGTATTCCTGTTGCTGTCTTCAGTCTCACAGAGAAGCTCCTGCTACTTCCCCAGCTCTCAGTAGTTTAGTAC	GTPase	Cell membrane and cytoplasm	Oncogene encoding a membrane protein that shuttles between the Golgi apparatus and the plasma membrane; mutations associated with colorectal cancers and follicular thyroid cancer	Driver Gene, MAPK, PI3K, RAS	1.69
*EIF2B4*, eukaryotic translation initiation factor 2B, subunit 4 delta, Chr. 2	NM_172195.3 ATGTACCAGTGCTGGTTTGCTGTGAAACATACAAGTTCTGTGAGCGTGTGCAGACTGATGCCTTTGTCTCTAATGAGCTAGATGACCCTGATGATCTGCA	GTP exchange factor	Cytoplasm	Catalyzes the exchange of EIF2‐bound GDP for GTP; mutations are a cause of leukoencephalopathy and ovarioleukodystrophy	‐	−0.08
*NF1*, neurofibromin 1, Chr. 17	NM_000267.2 TACCAGATCCCACAGACTGATATGGCTGAATGTGCAGAAAAGCTATTTGACTTGGTGGATGGTTTTGCTGAAAGCACCAAACGTAAAGCAGCAGTTTGGC	GTPase‐activating protein (GAP)	Cytoplasm	Negative regulation of the RAS/ MAPK signal transduction pathway by accelerating the hydrolysis of Ras‐bound GTP; mutated in neurofibromatosis type 1 (von Recklinghausen syndrome), juvenile myelomonocytic leukemia, and Watson syndrome	Driver Gene, MAPK, RAS	−1.11
*BMP2*, bone morphogenetic protein 2, Chr. 20	NM_001200.2 TTAGGATAAGCAGGTCTTTGCACCAAGATGAACACAGCTGGTCACAGATAAGGCCATTGCTAGTAACTTTTGGCCATGATGGAAAAGGGCATCCTCTCCA	Secreted ligand of the TGF‐beta receptors	Cell membrane, cytoplasm, extracellular and nucleus	Activation of SMAD family transcription factors; plays a role in bone and cartilage development; mutations lead to skeletal anomalies with or without cardiac anomalies	HH, TGF‐B	−0.58
*FZD9*, alias CD349, frizzled class receptor 9, Chr. 7	NM_007197.2 CCGTGCCGGCCACCTGTGTGATCGCCTGCTACTTTTACGAACGCCTCAACATGGATTACTGGAAGATCCTGGCGGCGCAGCACAAGTGCAAAATGAACAA	7‐transmembrane domain protein	Cell membrane	Receptor for Wnt signaling pathway coupled to the beta‐catenin signaling pathway; mutations are linked to Williams syndrome phenotype	Wnt	0.03
*IFNA17*, alias *LEIF2C1*,* IFN‐alpha I*, interferon, alpha 17, Chr. 9	NM_021268.2 TGAGATGATCCAGCAGACCTTCAATCTCTTCAGCACAGAGGACTCATCTGCTGCTTGGGAACAGAGCCTCCTAGAAAAATTTTCCACTGAACTTTACCAG	Cytokine, signaling protein	Extracellular, nucleus, cytoplasm	Related to type I IFN signaling pathway, antiviral activity	JAK‐STAT, PI3K	0.017

The identified gene transcripts showed genome‐wide localization (including Chr. 1, 2, 7, 9, 17, and 20). The distribution of the differentially expressed gene transcripts across the genome is highlighted in a Manhattan plot (Fig. S3). Boxplot diagrams of the signature levels in the SN, AST, and MST are shown in Fig. 3B. Of note, the top‐ranked signature gene *IFNA17* identified with the LASSO procedure is not within the group of the 68 differentially expressed genes in SN vs. MST (Table S2.1).

### Molecular signature in relation to histopathology and clinical features

In the following, the gene expression weight of the obtained molecular signature was calculated for all samples. The expression values of the defined signature genes *NRAS*,* EIF2B*,* NF1*,* BMP2*,* FZD9*, and *IFNA17* were multiplied with the LASSO coefficients per sample. Transformed proportions of the signature expression weights are given in Table S1 (last column).

The SN samples had a ‘low’ expression value (< 0.4) with a mean value of 0.24 (range 0–0.54). Samples SN2, SN10, and SN17 formed the exception (> 0.4). These samples showed a compound histopathological architecture. SN2 had been excised from the arm of a 9‐year‐old boy, SN10 from the cervical area of a 39‐year‐old female patient, and SN17 from the shoulder area of a 32‐year‐old female patient.

Interestingly, the AST samples had intermediate levels of the signature in between the group of SN and MST (mean value 0.43, range 0.19–0.81). The samples AST1‐3 and AST7 showed a ‘high’ gene expression weight (> 0.4) with a compound histopathological architecture and a trend toward a more central localization on the body (upper leg, gluteal area, back, and abdomen; Table S1). The samples AST4‐6 and AST8‐10 with a ‘low’ gene expression weight (< 0.4) showed localization on the extremities and central localization on the body. The AST samples with a ‘low’ gene expression weight had a dermal or compound histopathological architecture (Table S1). There was no significant difference in histopathological features such as mitotic index, or grade of nuclear atypia in between the ‘low’ and ‘high’ AST gene expression groups. All AST samples showed maturation within the dermal depth (data not shown).

The MST cases had a ‘high’ gene expression value (> 0.4) with a mean expression weight of 0.66 with exception of case MST4 (=0.38). There was a trend toward a higher Breslow thickness (mm) in association with the expression weight of the molecular signature; however, this failed to be statistically significant.

### Molecular signature in relation to public datasets

In Table S5, mean expression values and *P*‐values of the identified signature genes are shown for melanoma samples from the TCGA dataset [19]. The signature genes were significantly differentially expressed (*P* > 0.05) in primary vs. metastatic melanomas. The only exception was the gene transcript *BMP2* with a *P*‐value of 0.079. The *IFNA17* transcript showed no expression in the primary melanoma group, and little expression in the metastatic samples when compared to absolute expression values from the other transcripts. The *FZD9* transcript showed the highest range (1.10) in primary melanoma samples in relation to metastatic samples.

## Discussion

In the present study, we identified genes that are differentially expressed in the three categories of Spitzoid melanocytic neoplasms (SN, AST, and MST). Although some molecular studies have been performed on Spitzoid tumors, the transcriptome of these distinct lesions remains largely unknown to date [12, 13, 15. Therefore, the aim of this study was to identify a discriminating expression profile of SN, AST, and MST that would allow to discriminate these lesions from each other, and to define a list of genes that would add new insights into the underlying molecular basis of these lesions.

We performed the analysis using the PanCancer Pathways Panel from NanoString nCounter with a probe set of 770 genes related to cancer, growth, and invasion. NanoString nCounter gene expression profiling has been used to identify RNA expression patterns to accurately diagnose and classify different tumor types from various body sites [21, 22, 23. This platform does not require reverse transcription or amplification, and the measurement of gene expression at the RNA level yields information on the actual functional state of a cell. Moreover, it allows robust gene expression profiling from FFPE tissue. However, despite the large number of more than 700 investigated genes the gene selection represents a systematic bias. The PanCancer Pathways Panel is focused on intrinsic tumoral characteristics. Thus, (micro)environmental tumoral features such as inflammation and immune response might be underexplored in our study. It might be worthwhile to address Spitzoid melanocytic lesions with the PanCancer Immune Profiling Panel in future studies.

The three studied groups showed differential gene expression with 68 significant genes in the group SN vs. MST, 167 significant genes when SN were compared with AST, and 18 significant genes in AST vs. MST. The reason why the number of differentially expressed genes differs so much in the three studied conditions might be due to the applied statistical methodology. The group AST vs. MST showed the lowest sample size (*n* = 10 for AST and *n* = 14 for MST), and thus, differentially expressed genes may have failed to overcome the applied level of statistical significance (FDR *q*‐value with a threshold of 25%). However, the *limma* and *voom* methods were applied in this study to test for differential gene expression, which makes the analysis robust even when the sample size is small [17]. Another more likely explanation might be a higher genetic similarity (and thus a lower level of difference in the genetic make‐up) between AST and MST than between AST and banal SN. This explanation also reflects the experience from histopathological observation where the morphological differentiation between high‐grade AST and MST remains extremely challenging [5, 7, 8.

We identified a molecular gene expression signature including the six top‐ranked gene transcripts *NRAS*, *NF1*,* EIF2B4*,* IFNA17*, *BMP2*, and *FZD9* that could distinguish SN from MST. The gene transcripts *NF1* and *NRAS* are associated with the Ras/ Raf/ MEK/ ERK (MAPK) pathway, which is well known to be deregulated in melanoma development and pathogenesis of Spitzoid neoplasms [19, 24, 25, 26. Additionally, *NRAS* also plays an important role in the PI3/AKT (AKT) pathway [27, 28. We detected upregulation of *NRAS* transcripts, whereas *NF1* transcripts showed to be downregulated in SN vs. MST. The observed reciprocal expression is reasonable from a functional point of view because *NF1* encodes for neurofibromin, which stimulates the GTPase activity of *NRAS*, thereby inhibiting MAPK signaling [29, 30, 31. The oncogenic activation of *NRAS* has been reported in 15–25% of classical cutaneous melanomas [19] and has also been found to be alternated in the subgroup of Spitzoid melanocytic neoplasms [10, 32, 33. *NF1* alterations are found in approximately 10 % of cutaneous melanomas [19] and have also been reported in subgroups of Spitzoid melanocytic neoplasms [25, 34.

The identified signature gene *EIF2B4* encodes for eukaryotic initiation factor 2 (EIF2), which is composed of five different subunits and catalyzes the exchange of EIF2‐bound GDP for GTP [35]. The protein encoded by this gene is the fourth, or delta, subunit and has been associated with leukodystrophic neurodegenerative disease [36, 37, 38, 39. To our knowledge, there are no reports in the literature yet which have linked *EIF2B4* to melanocyte pathology.

Although *IFNA17* was not within the group of the 68 differentially expressed genes in SN vs. MST identified by the LASSO procedure, in combination with the other transcripts *NRAS*, *NF1*,* BMP2*,* EIF2B4*, and *FZD9*, it qualified as one of the top 6‐ranked signature genes. *IFNA17* encodes for the interferon (IFN)‐alpha 17 molecule and plays a role in the JAK/ STAT/ phosphatidylinositol 3'‐kinase (PI3K) pathway [40], which has also been reported to be deregulated in melanocytic neoplasms [41, 42. At the molecular level, it is well known that the IFNs have potent immune modulating and antitumor impacts on melanoma [43, 44. For decades, IFN‐alpha 2a/b has been the only approved drug for melanoma patients in the adjuvant setting [45]. However, different randomized trials have reported conflicting results about the therapeutic benefit [46, 47, 48 and the development of targeted therapies and discovery of new immunomodulating drugs have largely replaced adjuvant therapy in melanoma patients with IFN‐alpha [49]. Of note, the *IFNA17* gene is localized on Chr. 9p21.3 (start at 21 227 243 bp and end at 21 228 222 bp). The *p16* gene region encoding for cyclin‐dependent kinase inhibitor 2A is also localized on Chr. 9p21.9 (start 21 967 753 bp and end at 21 995 301) almost adjacent to the *IFNA17* gene. Alterations of the short arm of Chr. 9p21, especially homozygous deletions with loss of *p16* function, are associated with an aggressive clinical behavior in Spitzoid melanocytic lesions [50, 51, 52, 53. Thus, the observed lower expression of the *IFNA17* gene in the MST group compared to the SN group gives further evidence for transcriptional deregulation at this chromosomal locus in malignant Spitzoid lesions.

The *BMP2* transcript encodes for bone morphogenetic proteins (BMPs), a group of signaling molecules that are part of TGF‐β signaling [54], which has been reported to stimulate tyrosine gene expression and melanogenesis [55]. One may speculate that downregulation of *BMP2* may relate to the low levels of pigmentation, often observed in SN. To our knowledge, there are no reports in the literature yet that have linked *BMP2* to distinct categories of Spitzoid neoplasms yet.

Finally, the *FZD9* gene also qualified as one of the top‐ranked six transcripts for the gene signature and was upregulated in SN compared to MST. The transcript encodes for a frizzled class receptor (FZD) transmembrane receptor family member, which are part of the Wnt/ beta‐catenin signaling pathway [56, 57. The Wnt/ beta‐catenin pathway is well known to play a role in both development of melanocytes and their transformation to melanoma [58, 59, 60. The major effect of Wnt ligand binding to its receptor is the stabilization of cytoplasmic beta‐catenin through inhibition of the beta‐catenin degradation complex. Beta‐catenin is then free to enter the nucleus and activate Wnt‐regulated genes [61, 62. In line with *FZD9* upregulation, the *WIF1* transcript that encodes for Wnt inhibitory factor 1 (*WIF1*) was significantly downregulated with the lowest log FC of −1.17 from all 68 significantly differentially expressed genes in SN vs. MST. *WIF1* interacts with the Wnt protein and therefore negatively regulates receptor binding to the FZD receptor family that initiate Wnt/ beta‐catenin signaling [63]. When comparing our signature genes with the TCGA expression data, the *FZD9* transcript showed a higher mean expression value in primary cutaneous melanoma samples in relation to metastatic samples. A possible explanation is that *FZD9* may be a tumor suppressor in the presence of Wnt ligands in different kinds of cancer [64]. Our data on the *FZD9* and *WIF1* transcripts suggest an important role for the Wnt/ beta‐catenin pathway in Spitzoid lesions and deserve further study in a larger cohort of lesions.

The AST samples showed levels of the identified signature intermediate between the group of SN and the group of MST (Fig. 3B). This implies that the six gene expression signature can potentially be used to segregate low‐grade from high‐grade AST (those that share features of SN vs. those that are more similar to MST). Of note, histopathological features that are commonly taken as indicators of melanocytic malignancy, such as nuclear atypia, absence of maturation in the dermis, deep extension, and deep dermal mitoses, most often fail to differentiate benign from malignant potential in Spitzoid lesions [14, 65. Our findings are in line with this inconsistency. Although the identified signature could segregate ‘low’ and ‘high’ gene expression groups in the gray‐zone AST category, these two groups showed no significant differences in worrisome histopathological features when re‐examining cases. Thus, patients diagnosed with a gray‐zone AST category on histopathological grounds on forehand might receive more adopted treatment and ongoing follow‐up in the future in line with the underlying molecular biology of the lesion.

Our results indicate that the identified signature genes from our cohort are of relevance in several altered signal pathways in cutaneous melanoma such as reported by the TCGA group [19]. However, one should be careful to draw too meaningful conclusions from our comparative analysis with the dataset from the TCGA. First, despite the large number of melanoma samples in the TCGA SKCM dataset the majority of tissue for molecular analysis originated from metastatic sites (~ 80%, [19]). In contrast, in our series only the tissue from case MST4 was derived from a lymph node metastasis. All other tissue for molecular analysis yielded from the primary MST samples. Second, in the TCGA SKCM dataset 89% of the primary melanoma cases were obtained from advanced‐stage T4 melanomas with a Breslow thickness > 4 mm [19]. The authors reported that lower grade primary melanoma cases often had to be excluded from the investigation because there was not enough tissue available for further molecular analysis. In our series, the majority of MST represented lower stage melanomas (86%, *n* = 14, Breslow thickness between =/< 1.0 mm (T1) or 1.0–2.0 mm (T2); Table S1). Third, unfortunately the TCGA did not provide information about the histopathological subtypes of the investigated melanomas. Thus, a selection from the TCGA SKCM dataset of only Spitzoid melanoma subtypes for a comparative analysis with our cases was not possible.

In our study, the GSEA revealed downregulation of the Hallmark estrogen response early pathway and upregulation of the Hallmark spermatogenesis pathway in SN vs. MST (Fig. 2D). Indeed, eruptive SN have been documented during pregnancy and puberty, suggesting a potential role of hormonal deregulation in the pathogenesis of Spitz tumors [66]. Other case reports of disseminated SN also give hints to an endocrine pathogenesis with an affected patient suffering from Addison’s disease [67]. Thus, our data provide further evidence that hormonal alterations are worthwhile to be considered in the pathogenesis of Spitzoid melanocytic neoplasms.

The performed GSEA also revealed significant alterations in the Hallmark epithelial–mesenchymal transition pathway and the Hallmark coagulation pathway in the SN vs. AST and AST vs. MST conditions. Interestingly, in SN vs. AST there was significant upregulation of both pathways whereas in the group of AST vs. MST there was significant downregulation of these pathways. The same trend was seen for the Hallmark myogenesis pathway, the Hallmark angiogenesis pathway, and the Hallmark complement pathway with upregulation in the group of SN vs. AST and downregulation in the group of AST vs. MST. The opposing regulations of these gene sets again point to the intermediate state of AST between SN and MST.

Although the performed GSEA revealed no differential expression of gene pathways related to immunomodulatory and inflammatory processes, there were several differentially expressed immune‐related genes identifiable between the subgroups of Spitzoid melanocytic lesions (i.e., SN vs. AST vs. MST). These included *IL11RA*,* IL10*,* IL12RB2*,* IL11RB*,* IL13RA2*,* CD40*,* IL20RRB*,* IL7*,* IL20RA*,* LEF1*, and *IL1R2* (Table S2). In an earlier study, we have compared the gene expression profile of SN in relation to conventional NCN and observed significant upregulation of gene pathways with increased expression of transcripts related to immunomodulatory and inflammatory processes in the SN group [15]. Whereas NCN are rarely inflamed or hosted by immune cells, a reasonable subset of Spitzoid melanocytic lesions, independent from the SN, AST, or MST group, is accompanied by a prominent inflammatory infiltrate of immune cells [1, 68, 69. However, a more detailed characterization of this inflammatory microenvironment has not been performed yet. Interestingly, it is well known that similar to MST, AST frequently metastasize to locoregional lymph nodes, but in contrast to MST, rarely if ever spread to distant organs [6, 70. The mechanism behind this paradoxical behavior remains entirely unknown. As we have shown that there are only little molecular genetic differences in the tumor genome of the ambiguous AST category in comparison with the group of highly aggressive MST, one may speculate that the environmental immunologic neighborhood hosting the Spitzoid melanocytic neoplasms might define the lesion’s biological behavior with either arrest of metastatic potential in the locoregional lymph node or development of distant metastasis.

We are currently aiming to validate the identified top‐ranked gene transcripts of SN with immunohistochemistry to find out whether the signature is also discriminative on protein level. In the future, we also aim to validate this signature by NanoString nCounter gene expression analysis in a larger group of SN, AST, and MST to find out whether it can contribute to diagnosis and ongoing treatment in daily surgical pathology practice and clinical patient management.

## Conclusions

This is the first study employing transcriptomic NanoString nCounter analyses as a novel tool in the evaluation of challenging Spitzoid melanocytic neoplasms. We identified differentially expressed genes in the group of SN compared to MST and AST. A molecular gene expression signature comprising the six top‐ranked differentially expressed genes *NRAS*, *EIF2B4*,* NF1*,* BMP2*,* FZD9*, and *IFNA17* could differentiate in between SN and MST. The identified gene transcripts belong to important pathways involved in the development of melanocytic tumors and melanoma: the MAPK pathway, the AKT pathway, and the Wnt/ beta‐catenin pathway. Notably, AST samples showed intermediate levels of the signature, thereby implying that the six gene expression signature can potentially be used to distinguish more from less aggressive AST, thereby improving treatment decisions, and optimize clinical patient management in the future.

## Conflict of interest

The authors declare no conflict of interest.

## Author contributions

VW, JVO, AZH, and LH designed the research question and established the study. VW, JVO, MG, TG, and JB provided patient tissue from three medical centers. IS and JB performed the molecular genetic expression analysis. VW, JVO, MSG, and LH designed and developed the outline and concept of the manuscript. MSG and LH performed the bioinformatic analysis and edited the figures and tables. All authors wrote, discussed, and commented on the manuscript. All authors approved the final manuscript.

## Supporting information


**Fig**.** S1**. (A) Box‐plots of studied cases (51 samples) are shown including 27 Spitz nevi (SN), 10 atypical Spitz tumors (AST) and 14 malignant Spitz tumors (MST), that is, Spitzoid melanoma before (top) and after normalization (bottom) of data. (B) Multidimensionality scaling (MDS) plots of the data before (left) and after normalization (right).

**Fig**.** S2**. A heatmap of all the expression data (770 genes vs. 51 samples) is shown including 27 Spitz nevi (SN1‐27), 10 atypical Spitz nevi (AST1‐10) and 14 malignant Spitz tumors (MST1‐14), that is, Spitz melanoma, which is based on supervised clustering. The columns represent the 51 samples. Higher expression levels are shown in red and lower expression levels are shown in blue color (yellow staining represents an intermediate expression level).

**Fig**.** S3**. Manhattan plots for the three studied conditions with Spitz nevus (SN) vs. malignant Spitz tumor (MST), SN vs. atypical Spitz tumor (AST) and AST vs. MST. Data on chromosomal coordinates were from the University of Santa Cruz (UCSC) Genome Browser (version hg19). The name of the most significant gene per chromosome is shown.

**Table S1**. Patient Cohort with Clinical Data, Histopathological Features and Expression Weights of the Molecular Signature.
**Table S2**. Differentially Expressed Genes in SN vs. MST (S2.1), in SN vs. AST (S2.2) and in AST vs. MST (S2.3).
**Table S3**. Library size of the studied cases (SN, AST and MST).
**Table S4**. Characteristics of Identified Gene Pathways.
**Table S5**. Gene expression values of the signature genes in the Skin Cutaneous Melanoma (SKCM) dataset provided by the Cancer Genome Atlas (TCGA, [19]).

## Data Availability

The mRNA data are deposited in the NCBI’s Gene Expression Omnibus database (accession number: GSE139314).

## References

[1] Requena C , Requena L , Kutzner H and Sanchez Yus E (2009) Spitz nevus: a clinicopathological study of 349 cases. Am J Dermatopathol 31, 107–116. 19318795 10.1097/DAD.0b013e3181934218

[2] Spitz S (1948) Melanomas of childhood. Am J Pathol 24, 591–609. 18859360 PMC1942798

[3] Weedon D and Little JH (1978) The Spitz naevus. Aust N Z J Surg 48, 21–22. 276344 10.1111/j.1445-2197.1978.tb05798.x

[4] WHO (2018) Classification of Skin Tumours, 4th edn World Health Organization, WHO Press, Lyon.

[5] Mones JM and Ackerman AB (2004) "Atypical" Spitz's nevus, "malignant" Spitz's nevus, and "metastasizing" Spitz's nevus: a critique in historical perspective of three concepts flawed fatally. Am J Dermatopathol 26, 310–333. 15249862 10.1097/00000372-200408000-00008

[6] Lallas A , Kyrgidis A , Ferrara G , Kittler H , Apalla Z , Castagnetti F , Longo C , Moscarella E , Piana S , Zalaudek I *et al* (2014) Atypical Spitz tumours and sentinel lymph node biopsy: a systematic review. Lancet Oncol 15, e178–e183. 24694641 10.1016/S1470-2045(13)70608-9

[7] Cerroni L , Barnhill R , Elder D , Gottlieb G , Heenan P , Kutzner H , LeBoit PE , Mihm M Jr , Rosai J and Kerl H (2010) Melanocytic tumors of uncertain malignant potential: results of a tutorial held at the XXIX Symposium of the International Society of Dermatopathology in Graz, October 2008. Am J Surg Pathol 34, 314–326. 20118771 10.1097/PAS.0b013e3181cf7fa0

[8] McCormack CJ , Conyers RK , Scolyer RA , Kirkwood J , Speakman D , Wong N , Kelly JW and Henderson MA (2014) Atypical Spitzoid neoplasms: a review of potential markers of biological behavior including sentinel node biopsy. Melanoma Res 24, 437–447. 24892957 10.1097/CMR.0000000000000084

[9] Tetzlaff MT , Reuben A , Billings SD , Prieto VG and Curry JL (2017) Toward a molecular‐genetic classification of Spitzoid neoplasms. Clin Lab Med 37, 431–448. 28802494 10.1016/j.cll.2017.05.003

[10] Wiesner T , Kutzner H , Cerroni L , Mihm MC Jr , Busam KJ and Murali R (2016) Genomic aberrations in spitzoid melanocytic tumours and their implications for diagnosis, prognosis and therapy. Pathology 48, 113–131. 27020384 10.1016/j.pathol.2015.12.007 PMC4817351

[11] Hillen LM , Van den Oord J , Geybels MS , Becker JC , Zur Hausen A and Winnepenninckx V (2018) Genomic landscape of Spitzoid neoplasms impacting patient management. Front Med 5, 344. 10.3389/fmed.2018.00344 PMC6300473 30619857

[12] Jansen B , Hansen D , Moy R , Hanhan M and Yao Z (2018) Gene expression analysis differentiates melanomas from Spitz Nevi. J Drugs Dermatol 17, 574–576. 29742191

[13] Wu G , Barnhill RL , Lee S , Li Y , Shao Y , Easton J , Dalton J , Zhang J , Pappo A and Bahrami A (2016) The landscape of fusion transcripts in spitzoid melanoma and biologically indeterminate spitzoid tumors by RNA sequencing. Mod Pathol 29, 359–369. 26892443 10.1038/modpathol.2016.37 PMC4811687

[14] Raghavan SS , Peternel S , Mully TW , North JP , Pincus LB , LeBoit PE , McCalmont TH , Bastian BC and Yeh I (2020) Spitz melanoma is a distinct subset of spitzoid melanoma. Mod Pathol 1, 1–13. 10.1038/s41379-019-0445-z PMC7286778 31900433

[15] Hillen LM , Geybels MS , Rennspiess D , Spassova I , Ritter C , Becker JC , Garmyn M , Zur Hausen A , Van den Oord J and Winnepenninckx V (2018) Molecular profiling of Spitz nevi identified by digital RNA counting. Melanoma Res 28, 510–520. 30095598 10.1097/CMR.0000000000000495 PMC6221391

[16] Green R , Wilkins C , Thomas S , Sekine A , Ireton RC , Ferris MT , Hendrick DM , Voss K , de Villena FP , Baric R *et al* (2016) Identifying protective host gene expression signatures within the spleen during West Nile virus infection in the collaborative cross model. Genom Data 10, 114–117. 27843766 10.1016/j.gdata.2016.10.006 PMC5097955

[17] Ritchie ME , Phipson B , Wu D , Hu Y , Law CW , Shi W and Smyth GK (2015) limma powers differential expression analyses for RNA‐sequencing and microarray studies. Nucleic Acids Res 43, e47. 25605792 10.1093/nar/gkv007 PMC4402510

[18] Law CW , Chen Y , Shi W and Smyth GK (2014) voom: precision weights unlock linear model analysis tools for RNA‐seq read counts. Genome Biol 15, R29. 24485249 10.1186/gb-2014-15-2-r29 PMC4053721

[19] Cancer Genome Atlas Network (2015) Genomic classification of cutaneous melanoma. Cell 161, 1681–1696. 26091043 10.1016/j.cell.2015.05.044 PMC4580370

[20] Wu D and Smyth GK (2012) Camera: a competitive gene set test accounting for inter‐gene correlation. Nucleic Acids Res 40, e133. 22638577 10.1093/nar/gks461 PMC3458527

[21] Garcia PL , Miller AL , Kreitzburg KM , Council LN , Gamblin TL , Christein JD , Heslin MJ , Arnoletti JP , Richardson JH , Chen D *et al* (2016) The BET bromodomain inhibitor JQ1 suppresses growth of pancreatic ductal adenocarcinoma in patient‐derived xenograft models. Oncogene 35, 833–845. 25961927 10.1038/onc.2015.126 PMC6713275

[22] Braso‐Maristany F , Filosto S , Catchpole S , Marlow R , Quist J , Francesch‐Domenech E , Plumb DA , Zakka L , Gazinska P , Liccardi G *et al* (2016) PIM1 kinase regulates cell death, tumor growth and chemotherapy response in triple‐negative breast cancer. Nat Med 22, 1303–1313. 27775704 10.1038/nm.4198 PMC5552044

[23] Suzuki Y , Ng SB , Chua C , Leow WQ , Chng J , Liu SY , Ramnarayanan K , Gan A , Ho DL , Ten R *et al* (2017) Multiregion ultra‐deep sequencing reveals early intermixing and variable levels of intratumoral heterogeneity in colorectal cancer. Mol Oncol 11, 124–139. 28145097 10.1002/1878-0261.12012 PMC5527459

[24] Krauthammer M , Kong Y , Bacchiocchi A , Evans P , Pornputtapong N , Wu C , McCusker JP , Ma S , Cheng E , Straub R *et al* (2015) Exome sequencing identifies recurrent mutations in NF1 and RASopathy genes in sun‐exposed melanomas. Nat Genet 47, 996–1002. 26214590 10.1038/ng.3361 PMC4916843

[25] Lazova R , Pornputtapong N , Halaban R , Bosenberg M , Bai Y , Chai H and Krauthammer M (2017) Spitz nevi and Spitzoid melanomas: exome sequencing and comparison with conventional melanocytic nevi and melanomas. Mod Pathol 30, 640–649. 28186096 10.1038/modpathol.2016.237 PMC5413430

[26] Fecher LA , Amaravadi RK and Flaherty KT (2008) The MAPK pathway in melanoma. Curr Opin Oncol 20, 183–189. 18300768 10.1097/CCO.0b013e3282f5271c

[27] Davies MA (2012) The role of the PI3K‐AKT pathway in melanoma. Cancer J 18, 142–147. 22453015 10.1097/PPO.0b013e31824d448c

[28] Deng W , Gopal YN , Scott A , Chen G , Woodman SE and Davies MA (2012) Role and therapeutic potential of PI3K‐mTOR signaling in de novo resistance to BRAF inhibition. Pigment Cell Melanoma Res 25, 248–258. 22171948 10.1111/j.1755-148X.2011.00950.x

[29] Bollag G , Clapp DW , Shih S , Adler F , Zhang YY , Thompson P , Lange BJ , Freedman MH , McCormick F , Jacks T *et al* (1996) Loss of NF1 results in activation of the Ras signaling pathway and leads to aberrant growth in haematopoietic cells. Nat Genet 12, 144–148. 8563751 10.1038/ng0296-144

[30] Scheffzek K , Ahmadian MR , Kabsch W , Wiesmuller L , Lautwein A , Schmitz F and Wittinghofer A (1997) The Ras‐RasGAP complex: structural basis for GTPase activation and its loss in oncogenic Ras mutants. Science 277, 333–338. 9219684 10.1126/science.277.5324.333

[31] Larribere L and Utikal J (2016) Multiple roles of NF1 in the melanocyte lineage. Pigment Cell Melanoma Res 29, 417–425. 27155159 10.1111/pcmr.12488

[32] Da Forno PD , Pringle JH , Fletcher A , Bamford M , Su L , Potter L and Saldanha G (2009) BRAF, NRAS and HRAS mutations in spitzoid tumours and their possible pathogenetic significance. Br J Dermatol 161, 364–372. 19438459 10.1111/j.1365-2133.2009.09181.x

[33] Fullen DR , Poynter JN , Lowe L , Su LD , Elder JT , Nair RP , Johnson TM and Gruber SB (2006) BRAF and NRAS mutations in spitzoid melanocytic lesions. Mod Pathol 19, 1324–1332. 16799476 10.1038/modpathol.3800653

[34] Farah M , Nagarajan P , Curry JL , Tang Z , Kim TB , Aung PP , Torres‐Cabala CA , Eterovic AK , Wargo JA , Prieto VG *et al* (2018) Spitzoid melanoma with histopathologic features of ALK gene rearrangement exhibiting ALK copy number gain: a novel mechanism of ALK activation in spitzoid neoplasia. Br J Dermatol 180, 404–408. 29897634 10.1111/bjd.16881

[35] Kashiwagi K , Takahashi M , Nishimoto M , Hiyama TB , Higo T , Umehara T , Sakamoto K , Ito T and Yokoyama S (2016) Crystal structure of eukaryotic translation initiation factor 2B. Nature 531, 122–125. 26901872 10.1038/nature16991

[36] Gungor O , Ozkaya AK , Hirfanoglu T , Dilber C and Aydin K (2015) A rare mutation in EIF2B4 gene in an epileptic child with vanishing white matter disease: a case report. Genet Couns 26, 41–46. 26043506

[37] Kanbayashi T , Saito F , Matsukawa T , Oba H , Hokkoku K , Hatanaka Y , Tsuji S and Sonoo M (2015) Adult‐onset vanishing white matter disease with novel missense mutations in a subunit of translational regulator, EIF2B4. Clin Genet 88, 401–403. 25600065 10.1111/cge.12554

[38] Huyghe A , Horzinski L , Henaut A , Gaillard M , Bertini E , Schiffmann R , Rodriguez D , Dantal Y , Boespflug‐Tanguy O and Fogli A (2012) Developmental splicing deregulation in leukodystrophies related to EIF2B mutations. PLoS One 7, e38264. 22737209 10.1371/journal.pone.0038264 PMC3380860

[39] Liu R , van der Lei HD , Wang X , Wortham NC , Tang H , van Berkel CG , Mufunde TA , Huang W , van der Knaap MS , Scheper GC *et al* (2011) Severity of vanishing white matter disease does not correlate with deficits in eIF2B activity or the integrity of eIF2B complexes. Hum Mutat 32, 1036–1045. 21560189 10.1002/humu.21535

[40] Dubois A , Francois C , Descamps V , Fournier C , Wychowski C , Dubuisson J , Castelain S and Duverlie G (2009) Enhanced anti‐HCV activity of interferon alpha 17 subtype. Virol J 6, 70. 19493343 10.1186/1743-422X-6-70 PMC2697159

[41] Kreis S , Munz GA , Haan S , Heinrich PC and Behrmann I (2007) Cell density dependent increase of constitutive signal transducers and activators of transcription 3 activity in melanoma cells is mediated by Janus kinases. Mol Cancer Res 5, 1331–1341. 18171991 10.1158/1541-7786.MCR-07-0317

[42] Hassel JC , Winnemoller D , Schartl M and Wellbrock C (2008) STAT5 contributes to antiapoptosis in melanoma. Melanoma Res 18, 378–385. 19011510 10.1097/CMR.0b013e32830ce7d7

[43] Raig ET , Jones NB , Varker KA , Benniger K , Go MR , Biber JL , Lesinski GB and Carson WE 3rd (2008) VEGF secretion is inhibited by interferon‐alpha in several melanoma cell lines. J Interferon Cytokine Res 28, 553–561. 18771339 10.1089/jir.2008.0118 PMC2988463

[44] Palmer KJ , Harries M , Gore ME and Collins MK (2000) Interferon‐alpha (IFN‐alpha) stimulates anti‐melanoma cytotoxic T lymphocyte (CTL) generation in mixed lymphocyte tumour cultures (MLTC). Clin Exp Immunol 119, 412–418. 10691911 10.1046/j.1365-2249.2000.01159.x PMC1905588

[45] Kirkwood JM , Strawderman MH , Ernstoff MS , Smith TJ , Borden EC and Blum RH (1996) Interferon alfa‐2b adjuvant therapy of high‐risk resected cutaneous melanoma: the Eastern Cooperative Oncology Group Trial EST 1684. J Clin Oncol 14, 7–17. 8558223 10.1200/JCO.1996.14.1.7

[46] Kirkwood JM , Ibrahim JG , Sondak VK , Richards J , Flaherty LE , Ernstoff MS , Smith TJ , Rao U , Steele M and Blum RH (2000) High‐ and low‐dose interferon alfa‐2b in high‐risk melanoma: first analysis of intergroup trial E1690/S9111/C9190. J Clin Oncol 18, 2444–2458. 10856105 10.1200/JCO.2000.18.12.2444

[47] Hauschild A , Weichenthal M , Rass K , Linse R , Ulrich J , Stadler R , Volkenandt M , Grabbe S , Proske U , Schadendorf D *et al* (2009) Prospective randomized multicenter adjuvant dermatologic cooperative oncology group trial of low‐dose interferon alfa‐2b with or without a modified high‐dose interferon alfa‐2b induction phase in patients with lymph node‐negative melanoma. J Clin Oncol 27, 3496–3502. 19433681 10.1200/JCO.2008.21.3892

[48] Eggermont AM , Suciu S , Rutkowski P , Kruit WH , Punt CJ , Dummer R , Sales F , Keilholz U , de Schaetzen G , Testori A *et al* (2016) Long term follow up of the EORTC 18952 trial of adjuvant therapy in resected stage IIB‐III cutaneous melanoma patients comparing intermediate doses of interferon‐alpha‐2b (IFN) with observation: Ulceration of primary is key determinant for IFN‐sensitivity. Eur J Cancer 55, 111–121. 26790144 10.1016/j.ejca.2015.11.014

[49] Schadendorf D , van Akkooi ACJ , Berking C , Griewank KG , Gutzmer R , Hauschild A , Stang A , Roesch A and Ugurel S (2018) Melanoma. Lancet 392, 971–984. 30238891 10.1016/S0140-6736(18)31559-9

[50] Harms PW , Hocker TL , Zhao L , Chan MP , Andea AA , Wang M , Harms KL , Wang ML , Carskadon S , Palanisamy N *et al* (2016) Loss of p16 expression and copy number changes of CDKN2A in a spectrum of spitzoid melanocytic lesions. Hum Pathol 58, 152–160. 27569296 10.1016/j.humpath.2016.07.029

[51] Gammon B , Beilfuss B , Guitart J and Gerami P (2012) Enhanced detection of spitzoid melanomas using fluorescence in situ hybridization with 9p21 as an adjunctive probe. Am J Surg Pathol 36, 81–88. 21989344 10.1097/PAS.0b013e31822d5ff8

[52] Mason A , Wititsuwannakul J , Klump VR , Lott J and Lazova R (2012) Expression of p16 alone does not differentiate between Spitz nevi and Spitzoid melanoma. J Cutan Pathol 39, 1062–1074. 23005921 10.1111/cup.12014

[53] Gerami P , Cooper C , Bajaj S , Wagner A , Fullen D , Busam K , Scolyer RA , Xu X , Elder DE , Abraham RM *et al* (2013) Outcomes of atypical spitz tumors with chromosomal copy number aberrations and conventional melanomas in children. Am J Surg Pathol 37, 1387–1394. 23797719 10.1097/PAS.0b013e31828fc283

[54] Wang RN , Green J , Wang Z , Deng Y , Qiao M , Peabody M , Zhang Q , Ye J , Yan Z , Denduluri S *et al* (2014) Bone Morphogenetic Protein (BMP) signaling in development and human diseases. Genes Dis 1, 87–105. 25401122 10.1016/j.gendis.2014.07.005 PMC4232216

[55] Bilodeau ML , Greulich JD , Hullinger RL , Bertolotto C , Ballotti R and Andrisani OM (2001) BMP‐2 stimulates tyrosinase gene expression and melanogenesis in differentiated melanocytes. Pigment Cell Res 14, 328–336. 11601654 10.1034/j.1600-0749.2001.140504.x

[56] Wang YK , Samos CH , Peoples R , Perez‐Jurado LA , Nusse R and Francke U (1997) A novel human homologue of the Drosophila frizzled wnt receptor gene binds wingless protein and is in the Williams syndrome deletion at 7q11.23. Hum Mol Genet 6, 465–472. 9147651 10.1093/hmg/6.3.465

[57] Karasawa T , Yokokura H , Kitajewski J and Lombroso PJ (2002) Frizzled‐9 is activated by Wnt‐2 and functions in Wnt/beta ‐catenin signaling. J Biol Chem 277, 37479–37486. 12138115 10.1074/jbc.M205658200

[58] Delmas V , Beermann F , Martinozzi S , Carreira S , Ackermann J , Kumasaka M , Denat L , Goodall J , Luciani F , Viros A *et al* (2007) Beta‐catenin induces immortalization of melanocytes by suppressing p16INK4a expression and cooperates with N‐Ras in melanoma development. Genes Dev 21, 2923–2935. 18006687 10.1101/gad.450107 PMC2049194

[59] Chien AJ , Moore EC , Lonsdorf AS , Kulikauskas RM , Rothberg BG , Berger AJ , Major MB , Hwang ST , Rimm DL and Moon RT (2009) Activated Wnt/beta‐catenin signaling in melanoma is associated with decreased proliferation in patient tumors and a murine melanoma model. Proc Natl Acad Sci USA 106, 1193–1198. 19144919 10.1073/pnas.0811902106 PMC2626610

[60] Dantonio PM , Klein MO , Freire M , Araujo CN , Chiacetti AC and Correa RG (2018) Exploring major signaling cascades in melanomagenesis: a rationale route for targetted skin cancer therapy. Biosci Rep 38, BSR20180511. 30166456 10.1042/BSR20180511 PMC6167501

[61] Zhan T , Rindtorff N and Boutros M (2017) Wnt signaling in cancer. Oncogene 36, 1461–1473. 27617575 10.1038/onc.2016.304 PMC5357762

[62] Anastas JN and Moon RT (2013) WNT signalling pathways as therapeutic targets in cancer. Nat Rev Cancer 13, 11–26. 23258168 10.1038/nrc3419

[63] Hsieh JC , Kodjabachian L , Rebbert ML , Rattner A , Smallwood PM , Samos CH , Nusse R , Dawid IB and Nathans J (1999) A new secreted protein that binds to Wnt proteins and inhibits their activities. Nature 398, 431–436. 10201374 10.1038/18899

[64] Ueno K , Hirata H , Hinoda Y and Dahiya R (2013) Frizzled homolog proteins, microRNAs and Wnt signaling in cancer. Int J Cancer 132, 1731–1740. 22833265 10.1002/ijc.27746 PMC3940357

[65] Gerami P , Busam K , Cochran A , Cook MG , Duncan LM , Elder DE , Fullen DR , Guitart J , LeBoit PE , Mihm MC Jr *et al* (2014) Histomorphologic assessment and interobserver diagnostic reproducibility of atypical spitzoid melanocytic neoplasms with long‐term follow‐up. Am J Surg Pathol 38, 934–940. 24618612 10.1097/PAS.0000000000000198

[66] Onsun N , Saracoglu S , Demirkesen C , Kural YB and Atilganoglu U (1999) Eruptive widespread Spitz nevi: can pregnancy be a stimulating factor? J Am Acad Dermatol 40, 866–867. 10321637

[67] Ibsen HH and Clemmensen O (1990) Eruptive nevi in Addison's disease. Arch Dermatol 126, 1239–1240. 2396848 10.1001/archderm.126.9.1239

[68] Cesinaro AM , Foroni M , Sighinolfi P , Migaldi M and Trentini GP (2005) Spitz nevus is relatively frequent in adults: a clinico‐pathologic study of 247 cases related to patient's age. Am J Dermatopathol 27, 469–475. 16314701 10.1097/01.dad.0000185249.21805.d3

[69] Weedon D and Little JH (1977) Spindle and epithelioid cell nevi in children and adults. A review of 211 cases of the Spitz nevus. Cancer 40, 217–225. 880553 10.1002/1097-0142(197707)40:1<217::aid-cncr2820400134>3.0.co;2-2

[70] Hung T , Piris A , Lobo A , Mihm MC Jr , Sober AJ , Tsao H , Tanabe KK and Duncan LM (2013) Sentinel lymph node metastasis is not predictive of poor outcome in patients with problematic spitzoid melanocytic tumors. Hum Pathol 44, 87–94. 22939951 10.1016/j.humpath.2012.04.019

