# Supplementary figures and images for: A digital mRNA expression signature to classify challenging Spitzoid melanocytic neoplasms
Source: FEBS Open Bio. 2020 Jun 5;10(7):1326–41. doi: 10.1002/2211-5463.12897 (PMC7327909; doi:10.1002/2211-5463.12897)

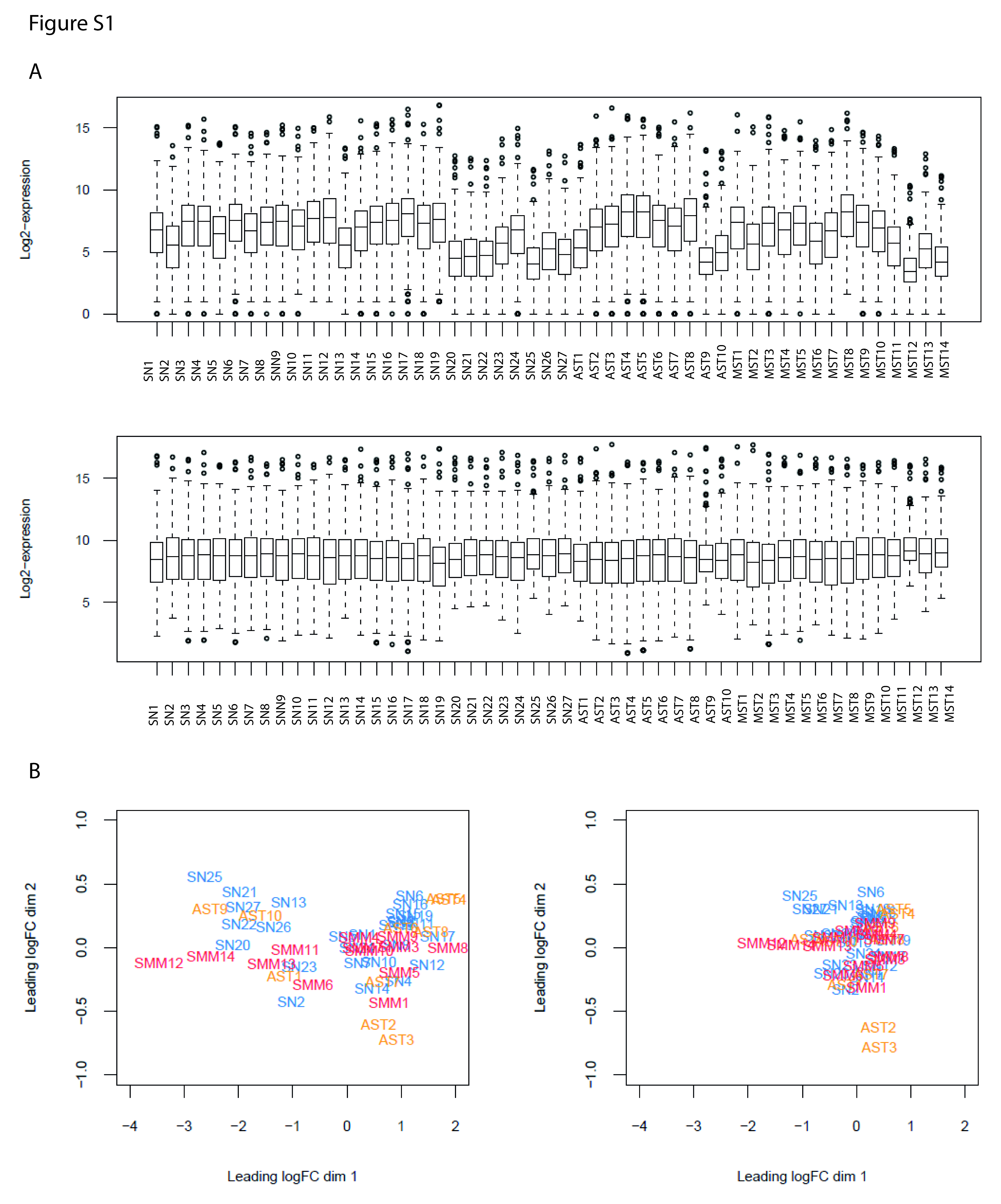

Supplement: Supplementary file 1 — Fig. S1. (A) Box‐plots of studied cases (51 samples) are shown including 27 Spitz nevi (SN), 10 atypical Spitz tumors (AST) and 14 malignant Spitz tumors (MST), that is, Spitzoid melanoma before (top) and after normalization (bottom) of data. (B) Multidimensionality scaling (MDS) plots of the data before (left) and after normalization (right). [file FEB4-10-1326-s001.tif]

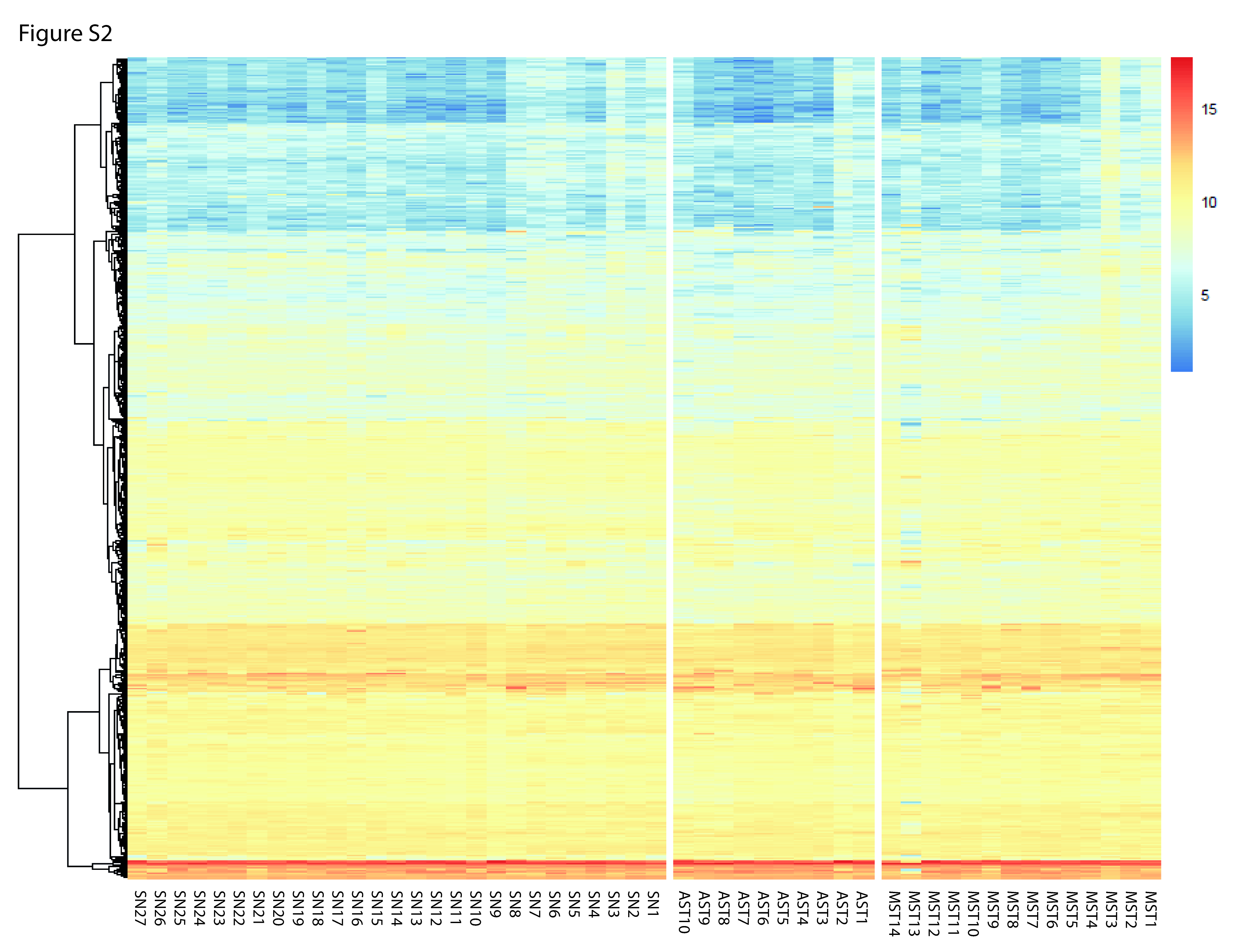

Supplement: Supplementary file 2 — Fig. S2. A heatmap of all the expression data (770 genes vs. 51 samples) is shown including 27 Spitz nevi (SN1‐27), 10 atypical Spitz nevi (AST1‐10) and 14 malignant Spitz tumors (MST1‐14), that is, Spitz melanoma, which is based on supervised clustering. The columns represent the 51 samples. Higher expression levels are shown in red and lower expression levels are shown in blue color (yellow staining represents an intermediate expression level). [file FEB4-10-1326-s002.tif]

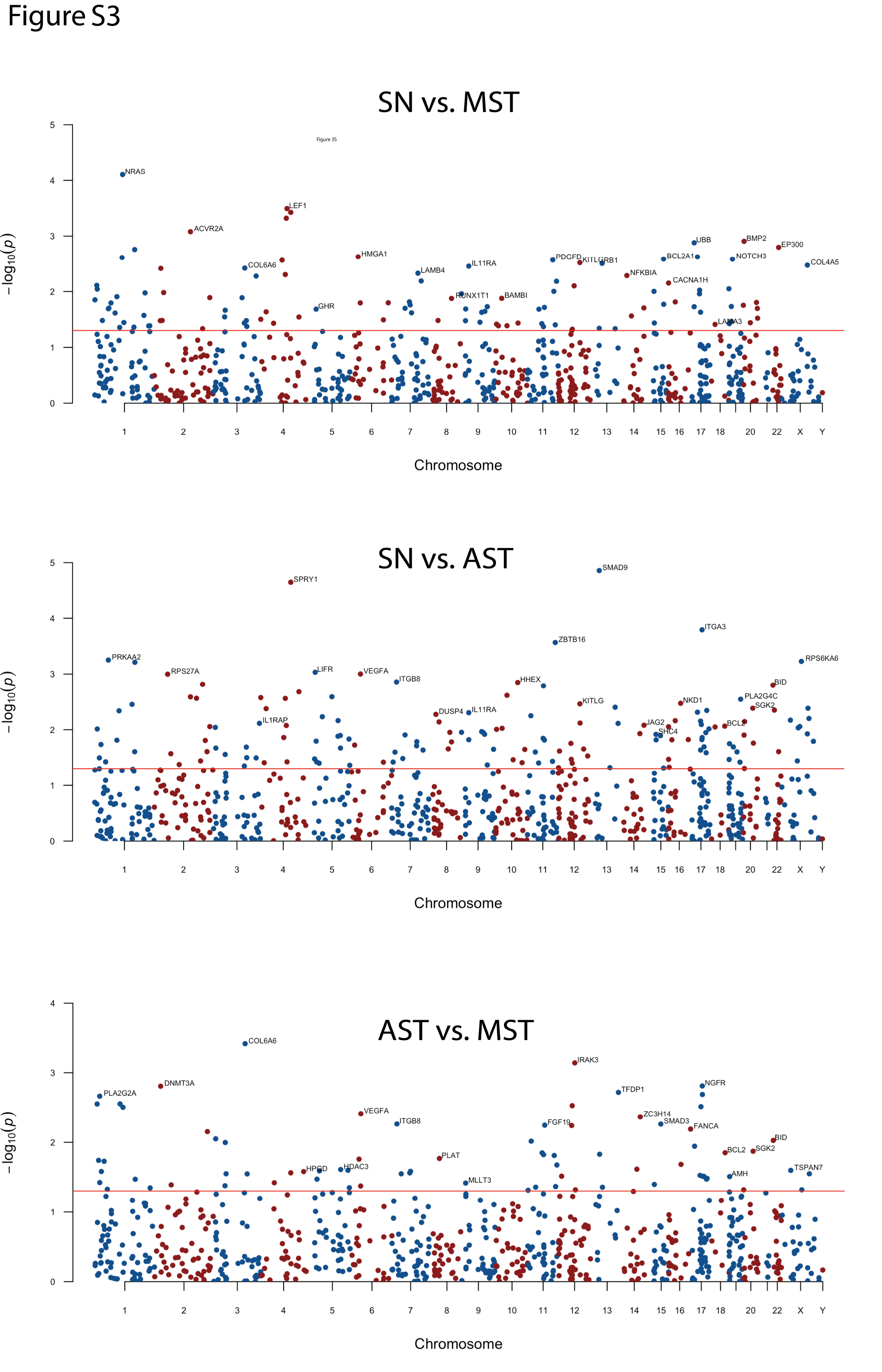

Supplement: Supplementary file 3 — Fig. S3. Manhattan plots for the three studied conditions with Spitz nevus (SN) vs. malignant Spitz tumor (MST), SN vs. atypical Spitz tumor (AST) and AST vs. MST. Data on chromosomal coordinates were from the University of Santa Cruz (UCSC) Genome Browser (version hg19). The name of the most significant gene per chromosome is shown. [file FEB4-10-1326-s003.png]
